# Imbalance of Th22/Treg cells causes microinflammation in uremic patients undergoing hemodialysis

**DOI:** 10.1042/BSR20191585

**Published:** 2019-10-11

**Authors:** Tingting Ren, Jingyuan Xiong, Guangliang Liu, Shaoyong Wang, Zhongqi Tan, Bin Fu, Ruilin Zhang, Xuesong Liao, Qirong Wang, Zonglin Guo

**Affiliations:** 1 The Eleventh People’s Hospital of Chengdu, Chengdu 610000, P.R. China; 2 West China School of Public Health and Healthy Food Evaluation Center, Sichuan University, Chengdu 610041, P.R. China

**Keywords:** Cytokines, Dialysis, Inflammation, T cell, Uremia

## Abstract

**Background:** Regulatory T (Treg) cells are of critical functionality in immune activation and inflammation in uremic patients undergoing hemodialysis (HD). A disruption in balance of Treg cells has potency to elicit infectious disease progression. Here, we examined possible association between ratio imbalance of Th22/Treg cells and microinflammation in uremic patients undergoing HD.

**Methods:** Peripheral blood mononuclear cells (PBMCs) were isolated to allow measurement of the percentage of Th22 cells and Treg cells using flow cytometry. Subsequently, serum levels of related cytokines, interleukin (IL) 22 (IL-22) and IL-10 and inflammatory factors, C-reactive protein (CRP), (TNF-α), IL-6 were determined via enzyme-linked immunosorbent assay (ELISA). Then relationships among dialysis time, microinflammation status (CRP) and dialysis adequacy (immunoreactive parathyroid hormone (iPTH), urea clearance index (K_t_/V), β2-MG, serum calcium, and serum phosphorus) were evaluated. Finally, correlation between microinflammation status and dialysis adequacy was analyzed with Pearson’s correlation coefficient.

**Results:** An increased percentage of Th22 and a decreased percentage of Treg cells were evident in uremic patients undergoing HD. Serum levels of IL-22, CRP, TNF-α, and IL-6 were increased, while IL-10 serum level was reduced. An imbalance of Th22/Treg cells was associated with microinflammation status in uremic patients undergoing HD. Furthermore, prolongation of the dialysis time, the microinflammation status and dialysis adequacy were changed. Increased dialysis adequacy was observed to correlate with alleviated microinflammation of uremic patients undergoing HD.

**Conclusions:** Conjointly, an imbalance of Th22/Treg cells may be a potential cause responsible for uremia occurrence, which in turn indicates that uremia could be effectively alleviated by altering the ratio of Th22/Treg cells.

## Introduction

Chronic kidney disease is a highly prevalent disease that commonly progresses to end-stage renal disease (ESRD) [1]. As a clinical disease commonly manifested by ESRD, uremia is characterized by metabolic abnormalities, electrolytes, and fluid as well as hormone imbalances contributing to the deterioration of renal function [2]. Multiple risk factors, including increased urea or creatinine, diabetes, hypertension, chronic inflammation, and even insulin resistance or dyslipidemia, have been identified to be associated with the occurrence and progression of uremia [2–5]. The therapeutic interventions approved in the clinical setting are limited to hemodialysis (HD), dialysis, and kidney transplantation, which incur with high mortality rates and poor long-term prognosis [6–8]. Inflammatory processes are clearly activated in patients at different stages of acute and chronic kidney disease, especially in patients undergoing HD [9]. An existing study has illustrated a vital link between acquired immunity disturbances in HD patients with T lymphocytes and antigen-presenting cells (APCs), with estimates of mortality induced due to inflammation to be 100–300-times higher in HD patients than the general population [10]. Thus, this raises concern for the development of more novel and accurate predictors to aid for an enhanced diagnostic modality and prognosis for uremia.

The novel T-helper cell subpopulations, such as T-helper type 1 (Th1), regulatory T (Treg) cells, and Th17, have been found to exercise certain effects on the immune balance, with reports exhibiting an unbalanced ratio of Treg and Th17 due to tissue inflammation, autoimmunity, and uremia [11,12]. Notably, CD4^+^ CD25^+^ Treg cells functioned as protective measure against renal injury in chronic renal disease by suppressing the activation of modulating macrophages, thereby reducing the production of proinflammatory cytokines and decreasing the effector phenotype [13]. Furthermore, the concentration of Treg cells was lower with impaired functionality in ESRD patients undergoing HD, and increased Treg cells could impede the progression of ESRD in patients via regulating the immune system [14]. Th22 cells, known as a distinct CD4 T-cell subset, could evidently secrete interleukin (IL) 22 (IL-22) independent of IL-17 and interferon (IFN), which was characterized by the aryl hydrocarbon receptor and chemokine receptors CCR4, CCR6 and CCR10 [15]. The Th22 cells induced by IL-22 were associated with increased inflammation in *Helicobacter pylori*-associated gastritis [16]. Additionally, increased number of Th22 and Th17 cells in the peripheral blood was found to extensively participate in the pathogenesis of inflammatory diseases [17]. With the aforementioned evidence serving as basis, we speculated the involvement of Th22 and Treg cells in uremia. Thus, the present study was planned to investigate the effects of Th22 and Treg cells on microinflammation in uremic patients with HD through the application of flow cytometry and enzyme-linked immunosorbent assay (ELISA) as well as evaluation of dialysis adequacy.

## Materials and methods

### Study subjects

A total of 146 patients (aged 36–70 years) with uremia who underwent treatment at the Eleventh People’s Hospital of Chengdu from February 2014 to February 2015 were recruited for the present study and then grouped into the dialysis group (82 patients receiving HD treatment) and the non-dialysis group (64 patients receiving no HD treatment). The inclusion criteria for the patients were as follows: (1) no acute infection in the previous 2 weeks; (2) no use of hormones and immunosuppressants in the past 6 months; and (3) no use of statins, levocarnitine, and thiazolidinedione hypoglycemic agents in the preceding month. The exclusion criteria for the patients were as follows: (1) a history of surgical intervention and trauma in the past 3 months; (2) presence of ischemic heart disease, cerebrovascular disease, blood disease, hepatitis, malignant weight, AIDS, and mental illness, or any other similar conditions; and (3) incomplete medical records. Meanwhile, a total of 42 healthy volunteers (aged 34–70 years) who had taken physical examination at the Eleventh People’s Hospital of Chengdu were selected as the control group. As shown in Table 1, no significant difference was evident in terms of age and sex among the three groups (*P*>0.05). In comparison with the control group, a reduced epidermal growth factor receptor (EGFR) expression was observed in the dialysis and non-dialysis groups, with similar observations in the dialysis group relative to the non-dialysis group (*P*<0.05).

**Table 1:** Comparison of general information among subjects in the three groups

Groups	M/F	Age (years)	Percentage of diabetic patients (%)	Chronic kidney disease etiology	Dialysis time (months)	EGFR (ml/min/1.73 m^2^)
Control	28/14	53.98 ± 8.66	0	No	0	95.75 ± 8.98
Dialysis	54/28	55.48 ± 9.07	35.37% (29/82)	Chronic glomerulonephritis and hypertensive nephropathy	11.60 ± 9.02	6.90 ± 2.21
Non-dialysis	40/24	52.88 ± 8.47	32.81% (21/64)	Chronic glomerulonephritis and hypertensive nephropathy	0	25.09 ± 3.06
F/c^2^	0.252	1.604	0.104			4855
*P*	0.882	0.204	0.747			<0.001

Abbreviations: ANOVA, analysis of variance; F, female; M, male. The control group, *n*=42; the dialysis group, *n*=82; the non-dialysis group, *n*=64. Measurement data are presented as mean ± standard deviation and analyzed using one-way ANOVA or Chi-square test.

### HD regimen

The patients in the non-dialysis group were administered a conservative drug treatment, while patients in the dialysis group received HD on the basis of drug treatment. Then the patients in the dialysis group were hemodialyzed through an internal arteriovenous fistula of the upper limb for two- to three-times every week (4–5 h/time, blood flow at 200–250 ml/min) over a period of at least 3 months. Additionally, the patients underwent dialysis using a polysulfone membrane dialyzer and a bicarbonate dialysate with a flow rate of 500 ml/min.

### Collection of blood samples and separation of peripheral blood mononuclear cells

Venous blood samples of 5–10 ml were collected from patients in fasting state admitted to the hospital in the early morning, which were then transferred to heparin anticoagulation tubes and heparin coagulation tubes. Thereafter, the mononuclear cells were isolated from the peripheral blood using Ficoll solution by means of density gradient equilibrium centrifugation. The cell suspension was prepared using 1640 culture medium with adjustment of the cell concentration to 3 × 10^6^ cells/ml. Following centrifugation, the supernatant was attained and preserved at −80°C for subsequent experimentation.

### Flow cytometry

Initially, the percentage of Treg cells in the peripheral blood was measured. Briefly, the peripheral blood mononuclear cells (PBMCs) were incubated with the CD4 antibodies (555346, BD Biosciences, Franklin Lakes, NJ, U.S.A.) and CD25 antibodies (551779, BD Biosciences, Franklin Lakes, NJ, U.S.A.) for surface staining. The cells were then subjected to staining with the forkhead box protein p3 (Foxp3)-phycoerythrine (PE) antibodies in the dark for 30 min. After a rinse with phosphate buffer saline (PBS), the cells were resuspended using 200 μl PBS and analyzed by conducting flow cytometry (DxFLEX6, Beckman Coulter Life Sciences, Brea, CA, U.S.A.). Subsequently, the percentage of Th22 cells in the peripheral blood was detected by flow cytometry. In short, CD3 monoclonal antibodies (563916, BD Biosciences, Franklin Lakes, NJ, U.S.A.) were added to the mononuclear cell suspension and cultured at 4°C for 20 min. Then, the cells were fixed, permeabilized, and incubated with the IL-22 monoclonal antibodies (AF782, R&D Systems, Minneapolis, MN, U.S.A.) at room temperature for 30 min according to the provided operating instructions. The mononuclear cells were rinsed with PBS once, resuspended using 200 μl PBS, and analyzed using flow cytometry (DxFLEX6, Beckman Coulter Life Sciences, Brea, CA, U.S.A.). Isotype controls were used as compensation controls and to confirm antibody specificity.

### ELISA

Samples were diluted in the ratio of 1:2 and the standard substances were diluted according to the necessitated ratios. The samples or standard substances (50 μl) were introduced in enzyme plates pre-embedded with the IL-22 (RAB1523), IL-10 (RAB0244), C-reactive protein (CRP, RAB0096), tumor necrosis factor-α (TNF-α, RAB1089), and IL-6 (RAB0306) antibodies. Biotinylated antibodies (50 μl/well) were added at room temperature for 2 h. The aforementioned antibodies were purchased from Sigma–Aldrich Chemical Company (St. Louis, MO, U.S.A.). Next, the plate was rinsed four times, supplemented with the streptavidin horseradish peroxidase solution (100 μl/well), incubated at room temperature for 1 h, and repeatedly rinsed four times. After the addition of a color-substrate solution (100 μl/well) for 10 min at room temperature in conditions devoid of light, the plate was supplemented with the termination solution (100 μl/well). Then, the optical density (OD) value was measured at an excitation wavelength of 450 nm. A standard curve was drawn and the contents of IL-22, IL-10, CRP, TNF-α, and IL-6 were determined.

### Evaluation of dialysis adequacy

The patients undergoing HD were divided into the 3-, 12-, and 24-month dialysis groups based on the dialysis time. Serum calcium and serum phosphorus were documented. The serum immunoreactive parathyroid hormone (iPTH) level was measured with radioimmunoassay (Medical System Biotechnology Co., Ltd., Zhejiang, China), and the β2-microglobulin (β2-MG) was determined by immunoturbidimetry in strict accordance with the provided instructions (Medical System Biotechnology Co., Ltd., Zhejiang, China). The urea clearance index (K_t_/V) was determined and calculated as follows: Kt/V = − In(R − 0.008 × t) + [(4 − 3.5 × R) × UF/W], where R = predialysis/postdialysis blood urea nitrogen (BUN), UF = predialysis − postdialysis weight change, W = postdialysis weight, and t = dialysis time. The urea reduction ratio (URR) was calculated as URP = 100 × (1 − Ct/Co). Ct was referred to as the urea level after dialysis and Co was a reference for the urea level before dialysis.

### Statistical analysis

All data analyses were processed using the SPSS 21.0 statistical software (IBM Corp., Armonk, NY, U.S.A.). Enumeration data were analyzed using the Chi-square test. Measurement data were expressed as mean ± standard deviation. All data conformed to normal distribution and homogeneity of variance. Comparisons between two groups were conducted using the unpaired *t* test while comparisons between multiple groups were assessed by one-way analysis of variance (ANOVA), followed by post hoc tests. Pearson’s correlation coefficient was adopted to determine the association between different parameters. A value of *P*<0.05 was considered to be statistically significant.

## Results

### An increased percentage of Th22 and a decreased percentage of Treg are found in the peripheral blood of uremic patients undergoing HD

**Figure 1:**
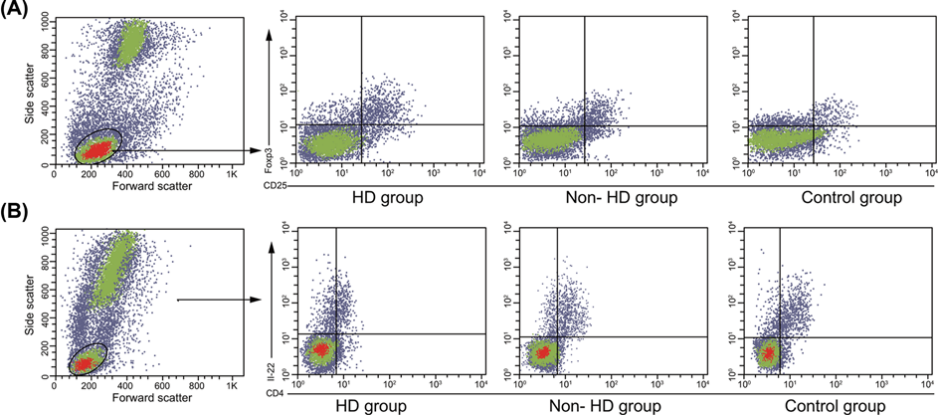
Percentage detection of Th22 and Treg cells in peripheral blood (**A**) The percentage of Th22 cells in peripheral blood detected using flow cytometry. (**B**) The percentage of Treg cells in peripheral blood detected using flow cytometry. Abbreviations: Th22, IL-22-producing helper T.

Initially, a regimen of flow cytometry was performed to detect the percentage of Th22 and Treg cells in peripheral blood. The results showed that the percentage of Th22 cells was higher in the dialysis group than that in the control and non-dialysis groups (*P*<0.05). The percentage of Treg cells was lower in the dialysis group than the percentage observed in the control and non-dialysis groups (*P*<0.05). In contrast with the dialysis group, the percentage of Th22 cells in the non-dialysis group was lower while that of Treg cells was higher. In comparison with the control group, the non-dialysis group illustrated an increased percentage of Th22 cells but a decreased percentage of Treg cells (Table 2 and Figure 1). These results suggested that uremic patients undergoing HD were demonstrative of a higher percentage of Th22 cells with a lower percentage of Treg cells in peripheral blood.

**Table 2:** Comparison of the percentage of Th22 and Treg cells in peripheral blood

Groups	Th22 (%)	Treg (%)
Control	0.46 ± 0.11	6.15 ± 1.24
Dialysis	3.67 ± 0.41*	1.63 ± 0.37*
Non-dialysis	1.64 ± 0.25^*†^	4.34 ± 0.64^*†^
F	1659	579.5
*P*	<0.001	<0.001

Abbreviation: Th22, T-helper type 22; T The control group, *n*=42; the dialysis group, *n*=82; the non-dialysis group, *n*=64. The data are presented as mean ± standard deviation, analyzed by one-way ANOVA. *P<0.05 *vs.* the control group. ^†^*P*<0.05 *vs.* the dialysis group.

### A higher concentration of IL-22 and a lower concentration of IL-10 exist in uremic patients undergoing HD

**Figure 2:**
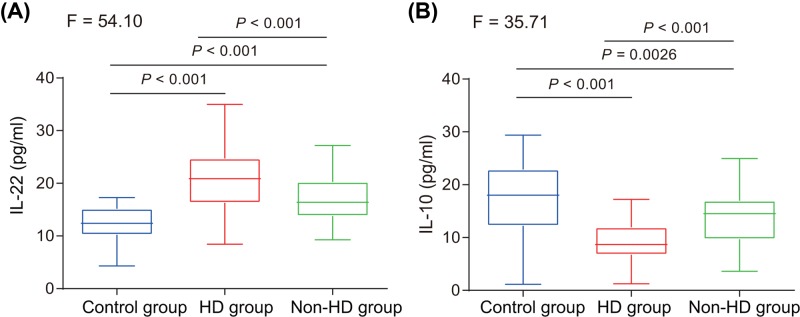
Concentration of IL-22 and IL-10 in uremic patients undergoing HD (**A**) The detection of IL-22 levels in plasma among the control, dialysis, and non-dialysis groups. (**B**) The detection of IL-10 levels in plasma among the control, dialysis, and non-dialysis groups; *P<0.05 *vs.* the control group; ^#^*P*<0.05 *vs.* the dialysis group. The data are presented as mean ± standard deviation, analyzed by one-way ANOVA.

Next, in order to detect plasma levels of IL-22 and IL-10 of uremic patients undergoing HD, ELISA was conducted. The results showed that, compared with the non-dialysis and control groups, the dialysis group had an increased concentration of IL-22 and a decreased concentration of IL-10 in the plasma. In comparison with the dialysis group, the concentration of IL-22 was lower in the non-dialysis group but higher in the control group, while that of IL-10 was higher in the non-dialysis group but lower in the control group (Table 3 and Figure 2). The aforementioned data revealed an increased concentration of IL-22 in combination with a decreased concentration of IL-10 in uremic patients undergoing HD.

**Table 3:** Levels of IL-22 and IL-10 in plasma of uremic patients undergoing HD (pg/ml)

Group	IL-22	IL-10
Control	12.13 ± 3.41	16.84 ± 7.12
Dialysis	20.83 ± 5.14*	9.19 ± 3.73*
Non-dialysis	16.82 ± 4.10^*†^	13.52 ± 4.61^*†^
F	54.10	35.71
*P*	<0.001	<0.001

The control group, *n*=42; the dialysis group, *n*=82; the non-dialysis group, *n*=64. Measurement data are presented as mean ± standard deviation, and analyzed by one-way ANOVA. *P<0.05 *vs.* the control group. ^†^*P*<0.05 *vs.* the dialysis group.

### High concentrations of CRP, TNF-α, and IL-6 are found in uremic patients

**Figure 3:**
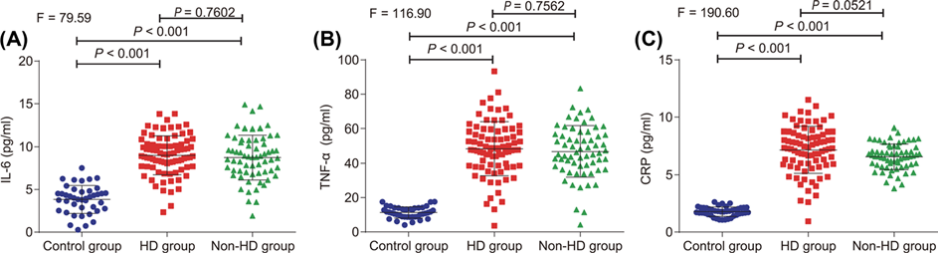
The concentrations of CRP, TNF-α, and IL-6 in uremic patients undergoing HD (**A**) The detection of IL-6 levels in serum among the control, dialysis, and non-dialysis groups. (**B**) The detection of TNF-α levels in serum among the control, dialysis, and non-dialysis groups. (**C**) The detection of CRP levels in serum among the control, dialysis, and non-dialysis groups; *P<0.05 *vs.* the control group.

ELISA was performed in an attempt to measure the levels of various inflammation-related factors (CRP, TNF-α, and IL-6) in serum, however no difference was evident in the serum levels of CRP, TNF-α, and IL-6 between the dialysis and non-dialysis groups (*P*>0.05). In comparison with the control group, the serum levels of CRP, TNF-α, and IL-6 were elevated in the dialysis and non-dialysis groups (*P*<0.05; Table 4 and Figure 3). The gathered findings suggested that uremic patients were evident of increased concentrations of CRP, TNF-α, and IL-6.

**Table 4:** Detection of the levels of inflammation-related factors (CRP, TNF-α, and IL-6) in serum

Group	IL-6 (pg/ml)	TNF-α (pg/ml)	CRP (pg/ml)
Control	3.84 ± 1.64	11.36 ± 3.25	1.79 ± 0.41
Dialysis	8.74 ± 2.64*	46.80 ± 14.87*	6.58 ± 1.12*
Non-dialysis	9.01 ± 2.28*	48.42 ± 15.64*	7.17 ± 2.03*
F	79.59	116.9	190.6
*P*	<0.001	<0.001	<0.001

The control group, *n*=42; the dialysis group, *n*=82; the non-dialysis group, *n*=64. Measurement data are presented as mean ± standard deviation and analyzed by one-way ANOVA. *P<0.05 *vs.* the control group.

### Imbalance of Th22/Treg cells relates to microinflammation status in uremic patients undergoing HD

**Figure 4:**
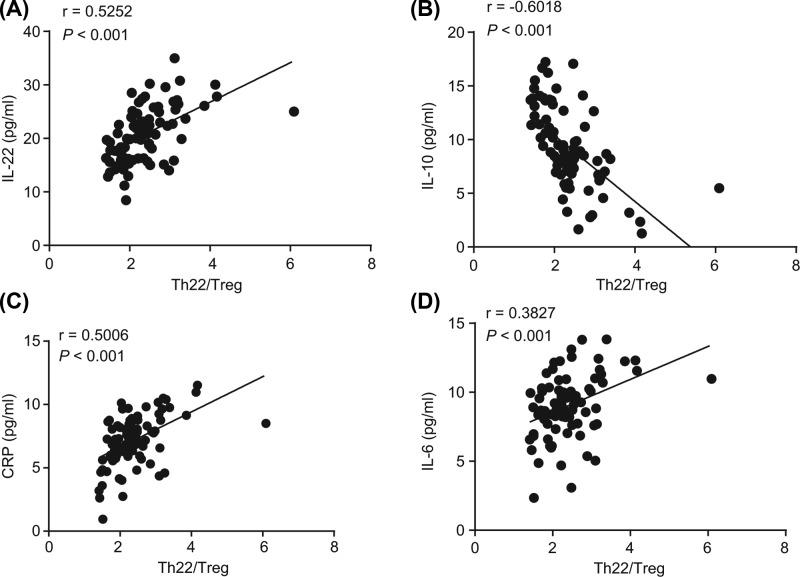
The microinflammation status of uremic patients is related to the imbalance of Treg/Th22 cells (**A**) Correlation analysis between IL-22 and Th22/Treg cells. (**B**) Correlation analysis between IL-10 and Th22/Treg cells. (**C**) Correlation analysis between CRP and Th22/Treg cells. (**D**) Correlation analysis between IL-6 and Th22/Treg cells. The results were recorded as measurement data and expressed as mean ± standard deviation. Pearson’s correlation coefficient was employed to determine the association between different parameters. Abbreviation: Th22, IL-22-producing helper T.

Subsequently, to investigate the relation between Th22/Treg cells and microinflammation in uremic patients undergoing HD, Pearson’s correlation coefficient was performed to conduct a subsequent correlation analysis. The results revealed that the Th22/Treg level in the supernatant of uremic patients undergoing HD was positively correlated with the cytokine IL-22 (Pearson’s correlation coefficient: *r* = 0.5252, *P*<0.05), while it was negatively correlated with IL-10 level (Pearson’s correlation coefficient: *r* = −0.6018, *P*<0.05). The level of CRP increased with an increased number of Th22/Treg cells and showed a linear trend (Pearson’s correlation coefficient: *r* = 0.5006, *P*<0.05). The level of IL-6 increased with the increase in Th22/Treg cells (Pearson’s correlation coefficient: *r* = 0.3827, *P*<0.05), that the levels of inflammatory cytokines CRP and IL-6 were positively correlated with Th22/Treg cell levels (Figure 4). Therefore, there were abnormal Th22/Treg cells and related cytokines in microinflammation of uremic patients who underwent HD.

### The microinflammation status and dialysis adequacy are changed with different dialysis time

To investigate the microinflammation status and dialysis adequacy of different dialysis times, the levels of CRP, iPTH, β2-MG, serum calcium, and serum phosphorus over different dialysis times were detected and compared. The levels of CRP, iPTH, β2-MG, and serum phosphorus were higher, while the levels of serum calcium in the 3-, 12-, and 24-month dialysis groups were lower than those in the control group (*P*<0.05). The levels of CRP, iPTH, β2-MG, serum phosphorus, and URR were lower, however the levels of serum calcium and K_t_/V were higher in the 24-month dialysis group compared with the levels observed in the 12- and 3-month dialysis groups (*P*<0.05). The levels of CRP, iPTH, β2-MG, serum phosphorus, and URR were lower, and the levels of serum calcium and K_t_/V in the 12-month dialysis group were higher than those in the 3-month dialysis group (*P*<0.05). The levels of CRP, iPTH, β2-MG, serum phosphorus, and URR were reduced, while the levels of serum calcium and K_t_/V were elevated over progression of the dialysis time (Table 5). The aforementioned findings suggested that prolonged dialysis time could effectively improve dialysis adequacy and prevent subsequent microinflammation in uremic patients receiving HD.

**Table 5:** Comparison of the microinflammation status and dialysis adequacy among different dialysis times

Group	CRP (ng/ml)	K_t_/V	iPTH (ng/l)	β2-MG (mg/l)	Serum phosphorus (mmol/ml)	Serum calcium (mmol/ml)	URR (%)
Control	1.79 ± 0.41	—	46.89 ± 5.41	2.41 ± 0.28	1.02 ± 0.17	2.35 ± 0.10	—
3-month	8.73 ± 1.36*	1.12 ± 0.19	533.42 ± 27.91*	9.04 ± 0.68*	2.20 ± 0.19*	1.73 ± 0.09*	67.25 ± 6.22
12-month	7.20 ± 1.80*	1.66 ± 0.28	478.41 ± 27.27*	6.93 ± 0.80*	1.91 ± 0.17*	1.93 ± 0.13*	56.30 ± 6.19
24-month	6.10 ± 1.88*	2.14 ± 0.19	352.07 ± 32.61*	4.71 ± 0.73*	1.48 ± 0.16*	2.11 ± 0.33*	51.01 ± 6.01
F	187.3	170.4	3103	821.6	325.9	87.41	55.90
*P*	<0.001	<0.001	<0.001	<0.001	<0.001	<0.001	<0.001

Abbreviations: β2-MG, β2-microglobulin. The control group, *n*=42; 3-month dialysis group, *n*=37; the 3-month dialysis group, *n*=20; the 24-month dialysis group, *n*=25. Measurement data are presented as mean ± standard deviation, and analyzed by one-way ANOVA. *P<0.05 *vs.* the control group.

### Dialysis inadequacy accelerates microinflammation in uremic patients undergoing HD

In our aforementioned study, the changes in dialysis adequacy, microinflammation status, and other clinical indicators in uremic patients undergoing HD at different time periods were observed, which were further ascertained by conducting a correlation analysis. The results showed that iPTH and β2-MG were positively correlated while K_t_/V was negatively correlated to CRP (Table 6). This further uncovered that dialysis adequacy was in a negative correlation with microinflammation.

**Table 6:** Correlation analysis among the iPTH, β2-MG, and Kt/V

Index		iPTH	β2-MG	K_t_/V
hCRP	r	0.524	0.524	−0.503
	*P*	<0.001	<0.001	<0.001

Abbreviations: hCRP, highly sensitive CRP; β2-MG, β2-microglobulin. Pearson correlation coefficient was used to determine the association between different parameters.

## Discussion

HD has been proven to be an efficacious therapeutic intervention for aiding the quality of life prolonging the survival of uremic patients by effectively reducing the amount of small molecular toxins [18]. However, uremia and its related treatment may result in immune changes and microinflammation in HD patients [19,20]. For instance, microinflammation in HD patients induces the expression of indoleamine 2,3-dioxygenase [21]. This enzyme can effectively down-regulate the T-cell receptor (TCR) ζ-chain [22], which has been documented in HD patients [23]. In recent years, Treg and Th17 cells, as a subclass of CD4^+^ T cells, were shown to be involved in inflammatory responses in uremic patients who underwent treatment with HD [24]. This study explored the potential functionality of a Treg/Th22 cell imbalance in microinflammation of uremic patients who underwent HD, and the results revealed that a Treg/Th22 cell imbalance participated in microinflammation that induced uremia progression in patients undergoing HD.

Initially, the data obtained in the present study demonstrated that the percentage of Th22 cells was increased and the percentage of Treg cells was decreased in PBMCs of uremic patients. The distribution of Th22 cells was shown to be increased in patients suffering from immune thrombocytopenia [25]. A previous study established a correlation between Th22 cells with a notably increased immune response and tissue inflammation [26]. Induced Treg cells were associated with elevated autoimmunity and susceptibility to inflammatory diseases [27]. In addition, research has observed the functional imbalance of Treg and Th17 cells to be linked with an increased incidence of immune-mediated cardiovascular complications in uremic patients on maintenance HD [28].

Furthermore, in our study, an imbalance of Treg/Th22 cells was found to induce abnormal microinflammation in uremic patients receiving HD, as supported by elevated levels of IL-22, CRP, TNF-α, IL-6, and a decreased IL-10 level among the PBMCs of patients treated with HD. Traditional inflammatory biomarkers, including CRP, TNF-α, and IL-6, have been found to be predictors of cardiovascular disease and even uremia [29]. A previous study reported that induced levels of IL-6, CRP, and carbonyl content were evident in ESRD patients undergoing HD [30]. IL-10, an anti-inflammatory cytokine has been reported to be involved in inflammation of uremic intoxication in dialysis patients [31]. Data showed that an overexpressed profile of the urinary IL-22 binding protein may result in inflammation among patients with active renal disease [32]. Lin et al. [33] asserted that IL-22, known to be a member of the IL-10 family of cytokines, was predominantly produced by Th22 cells and innate lymphocytes, and Treg cells were found to increase IL-22 levels during acute inflammation. Collectively, our results indicated that Treg and Th22 cells might be imperative targets for the treatment of uremia.

A previous study demonstrated the functionality of an elevated dialysis time to effectively improve dialysis adequacy in dialysis patients [34]. Consistently, our results also indicated that the microinflammation status and dialysis adequacy could be altered with different dialysis times. Maksic et al. [35] augmented the idea that chronic inflammation was the resultant of an increased dialysis time, as supported by increased serum levels of TNF-α and IL-6 in patients with chronic renal failure. In addition, a positive correlation between dialysis time and HD adequacy parameters was evident [36]. Thus, prolonged dialysis time could potentially improve dialysis adequacy and prevent subsequent microinflammation in uremic patients undergoing HD.

Significant improvements were evident in PTH, β2-MG, and CRP levels in ESRD patients undergoing HD [37]. β2-MG was positively correlated with CRP, which might be involved in the development of Henoch-Schönlein purpura [38]. In addition, a decreased level of CRP and β2-MG might help ameliorate persistent fever and destructive arthritis [39]. A positive correlation between PTH and CRP has been demonstrated in HD patients [40]. Rashid et al. [41] reported that low K_t_/V is an indicative of dialysis inadequacy, which is associated with chronic inflammatory state, resulting in high CRP levels. Findings obtained from a study flagged the association of reduced inflammation in peritoneal dialysis patients with elevated dialysis adequacy [42]. Furthermore, an inhibited microinflammation was crucial for improving the survival rates of uremic patients [20]. Consistent with our findings, iPTH and β2-MG were positively correlated with CRP while K_t_/V was negatively correlated with CRP. Therefore, we concluded that dialysis inadequacy can augment microinflammation in uremic patients undergoing HD.

In summary, our study revealed that the imbalance of Th22/Treg cells could potentially contribute to occurrence of uremia in patients following HD. However, there has been no further study on whether the Th22/Treg cell ratio can be altered and what impact the change in the Th22/Treg cell ratio could have on uremic patients undergoing HD. Hence, further study was warranted to analyze the specific role and functionality of Th22/Treg cells in uremic patients undergoing HD based on *in vivo* experiments.

## Abbreviations

CRP: C-reactive protein
ELISA: enzyme-linked immunosorbent assay
ESRD: end-stage renal disease
HD: hemodialysis
IL: interleukin
iPTH: immunoreactive parathyroid hormone
K_t/_V: urea clearance index
PBMC: peripheral blood mononuclear cell
PBS: phosphate buffer saline
Th1: T-helper type
TNF-α: tumor necrosis factor-α
Treg: regulatory T
URR: urea reduction ratio
